# Damage-Associated Molecular Patterns in Perioperative Anesthesia Care: A Clinical Perspective

**DOI:** 10.3390/anesthres3010001

**Published:** 2025-12-20

**Authors:** Wiriya Maisat, Koichi Yuki

**Affiliations:** 1 Department of Anesthesiology, Faculty of Medicine Siriraj Hospital, Mahidol University, Bangkok 10700, Thailand; 2 Department of Anesthesiology, Critical Care and Pain Medicine, Boston Children’s Hospital, Boston, MA 02115, USA; 3 Department of Anaesthesia and Immunology, Harvard Medical School, Boston, MA 02115, USA

**Keywords:** damage-associated molecular patterns, sterile inflammation, systemic inflammatory response syndrome, ischemia–reperfusion injury, organ dysfunction

## Abstract

Damage-associated molecular patterns (DAMPs) are endogenous molecules released during cellular stress or injury that trigger sterile inflammation. In perioperative settings, common triggers include surgical trauma, ischemia–reperfusion injury, cardiopulmonary bypass, blood transfusion, and mechanical ventilation. When released extracellularly, DAMPs activate innate immune receptors such as Toll-like receptors (TLRs) and the receptor for advanced glycation end products (RAGE), initiating signaling cascades that amplify inflammation, disrupt endothelial integrity, and promote coagulation and metabolic imbalance. This sterile inflammatory response may extend local tissue injury into systemic organ dysfunction, manifesting clinically as acute lung injury, acute kidney injury, myocardial dysfunction, disseminated intravascular coagulation, and perioperative neurocognitive disorders. Recognizing the central role of DAMPs reframes these complications as predictable consequences of endogenous danger signaling rather than solely as results of infection or hemodynamic instability. This understanding supports the use of established strategies such as protective ventilation and restrictive transfusion to minimize DAMP release. Emerging evidence also suggests that anesthetic agents may influence DAMP-mediated inflammation: propofol and dexmedetomidine appear to exert anti-inflammatory effects, whereas volatile anesthetics show variable results. Although clinical data remain limited, anesthetic choice and perioperative management may significantly affect systemic inflammatory burden and recovery. Future research validating DAMPs as biomarkers and therapeutic targets may inform precision anesthetic strategies aimed at modulating sterile inflammation, ultimately enhancing perioperative outcome.

## 1. Introduction

Anesthesiology has evolved far beyond the roles of inducing unconsciousness and analgesia. Modern anesthesiologists function as perioperative physicians, managing complex physiological challenges and preventing complications that may arise long after anesthesia ends. Despite improvement in surgical techniques, anesthesia management, monitoring, and perioperative care, postoperative complications such as acute lung injury (ALI), acute kidney injury (AKI), myocardial dysfunction, and delirium remain common and continue to affect patient recovery. Traditionally, these issues have been attributed to perioperative ischemia, hemodynamic instability, infection, or drug effects. However, emerging research suggests that damage-associated molecular patterns (DAMPs), molecules released endogenously as danger signals, may fundamentally contribute to the development of systemic inflammation and organ [1].

The concept of DAMPs originated from the recognition that inflammation can occur independently of infection [2]. These molecules are normally contained within cells but are released following cellular stress, injury, or necrosis. Once outside the cell, DAMPs are recognized mainly by the innate immune system as signals of tissue damage, provoking inflammatory responses that resembles sepsis, even in the absence of microbial infection.

Although the terminology around DAMPs may seem abstract or overly focused on molecular immunology, their clinical importance is direct and tangible. DAMPs are released during common perioperative events, such as surgical incisions, transfusions, ischemia followed by reperfusion, and the use of cardiopulmonary bypass (CPB). After their release, these molecules can initiate or amplify inflammation, contributing to postoperative complications. These mechanisms may also help explain why some patients develop systemic complications despite apparently uneventful intraoperative courses. Differences in both the extent of tissue injury and the individual’s immune response may influence postoperative trajectories.

This review provides a clinician-oriented perspective on DAMPs, with a focus on their perioperative sources, pathophysiological consequences, and relevance to anesthetic management. By translating emerging scientific knowledge into practical clinical understanding, we aim to offer anesthesiologists a practical framework to better anticipate, recognize, and potentially mitigate DAMP-related complications.

## 2. Methods

This narrative review was conducted to examine the role of DAMPs in perioperative inflammation and organ dysfunction, with an emphasis on their relevance to anesthesia care. A targeted literature search was performed across MEDLINE, PubMed, and Google Scholar for articles published up to September 2025. Primary search terms included: “*damage-associated molecular patterns*”, “*DAMPs*”, “*sterile inflammation*”, “*postoperative complications*”, and “*anesthesia*”. Additional keywords were combined to refine the search, such as “*ischemia–reperfusion injury*”, “*cardiopulmonary bypass*”, “*mechanical ventilation*”, “*transfusion*”, “*acute lung injury*”, “*acute kidney injury*”, “*myocardial dysfunction*”, and “*perioperative neurocognitive disorders*”.

To capture relevant mechanistic and translational insights, supplementary searches were performed for specific DAMP molecules, pattern recognition receptors, and inflammatory mediators. Eligible studies included basic science experiments, animal models, translational research, clinical trials, systematic reviews, and narrative reviews. Articles were selected based on their relevance to perioperative DAMP release, signaling, and pathophysiological consequences, as well as the influence of anesthetic agents and perioperative practices. Case reports, commentaries, and correspondence pieces were excluded. Reference lists of included articles were also screened to identify additional relevant studies. The initial search identified approximately 7930 articles, of which 130 were selected for inclusion in this review.

Given the narrative nature of this review, a formal systematic review methodology was not employed. As such, the possibility of selection bias and incomplete coverage of the literature remains a limitation of this work.

## 3. Understanding Damage-Associated Molecular Patterns (DAMPs)

DAMPs are endogenous molecules that are released from the body’s own cells when they are stressed, injured, or undergo unregulated forms of cell death [3]. Under normal conditions, these molecules remain confined within cells, where they play essential roles in metabolism, structural organization, and gene regulation. However, when they are released into the extracellular environment, they are detected by pattern recognition receptors (PRRs) on immune and non-immune cells, which perceive their presence as a sign of tissue injury. This interaction initiates a cascade of immune responses that can lead to sterile inflammation, even when no pathogens are present [4].

### 3.1. Historical Background and the Danger Model of Immunity

Consider a patient who develops systemic inflammatory response syndrome (SIRS) following major surgery, severe trauma, acute pancreatitis, or ischemic-reperfusion injury after organ transplantation. Despite the absence of identifiable infection, the patient presents with fever, hypotension, leukocytosis, and signs of multi-organ dysfunction. Blood cultures remain negative, and no microbial source is identified. The clinical presentation, however, closely resembles sepsis. Such scenarios have long challenged the traditional views that inflammation arises primarily in response to infection.

Earlier concepts in immunology proposed that the immune system functions primarily by distinguishing between *self* and *non-self*. In this model, immune responses were directed against foreign entities such as bacteria, viruses, or transplanted tissues, while endogenous components were presumed to be tolerated. This framework provided a useful basis for understanding host defense and transplant rejection. The subsequent discovery of pathogen-associated molecular patterns (PAMPs) added molecular details to the theory. PAMPs are conserved microbial structures absent from host cells, recognized by PRRs on immune cells, which then initiate protective responses to infection [5]. While this model clarified how the innate immune system detects pathogens, it did not adequately explain inflammation triggered by sterile tissue injury [6].

To address this gap, the “danger model” of immunity was proposed [7]. This model suggests that immune responses can be triggered not only by microbial components but also by endogenous signals released during cellular stress or injury [8,9]. These signals, now recognized as DAMPs, are normally contained within healthy cells. When released into the extracellular environment, they are perceived as misplaced and interpreted by PRRs as indicators of danger. Thus, immune activation reflects not only the detection of *non-self*, but also the recognition of tissue injury within *self*.

### 3.2. Perioperative DAMPs and Immune Recognition

The term *DAMP* refers to endogenous molecules that alert the immune system when they appear outside their normal intracellular locations. They are sometimes also called *alarmins*, a term that emphasizes their role as rapid alarm signals, often released actively by stressed or immune cells [10]. Although the terminology differs slightly, both concepts describe the same phenomenon, and in this review, we will use the term DAMPs for clarity.

**Figure 1.**
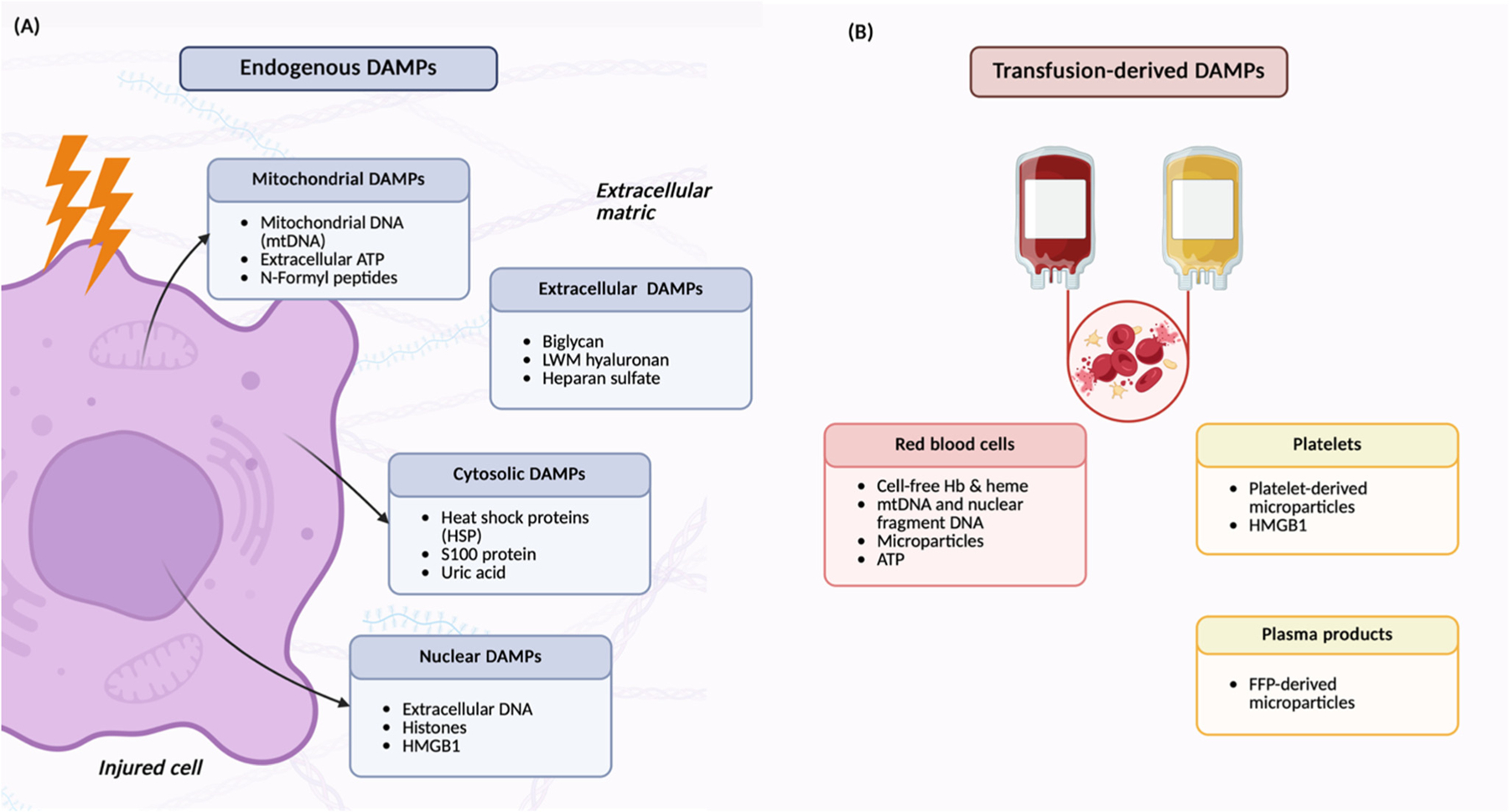
Schematic representation of the sources and types damage-associated molecular patterns (DAMPs). (**A**) Endogenous DAMPs released from an injured cell. The figure illustrates the major subcellular origins of DAMPs, including mitochondrial DAMPs (mitochondrial DNA, extracellular ATP, N-formyl peptides), cytosolic DAMPs (heat shock proteins, S100 proteins, uric acid), and nuclear DAMPs (extracellular DNA, histones, HMGB1). Elements of the extracellular matrix such as biglycan, low-molecular-weight hyaluronan, and heparan sulfate are also shown as extracellular-derived DAMPs. (**B**) Transfusion-derived DAMPs associated with different blood products. Red blood cell units may contain cell-free hemoglobin and heme, mitochondrial and nuclear DNA fragments, microparticles, and ATP. Platelet units may release platelet-derived microparticles and HMGB1. Plasma products, including fresh frozen plasma, may contain plasma-derived microparticles. Explanation in the text. Created in BioRender. Maisat, W. (2025) https://BioRender.com/7ldsmk0.

DAMPs include molecules with diverse structures and functions, such as nuclear proteins, mitochondrial components, cytosolic enzymes, extracellular matrix fragments, and transfusion-derived products [3,11] (Table 1, Figure 1). Within healthy cells they contribute to essential biological processes but once released into the extracellular space they are interpreted as signals of tissue injury and can provoke an immune response.

**Figure 2.**
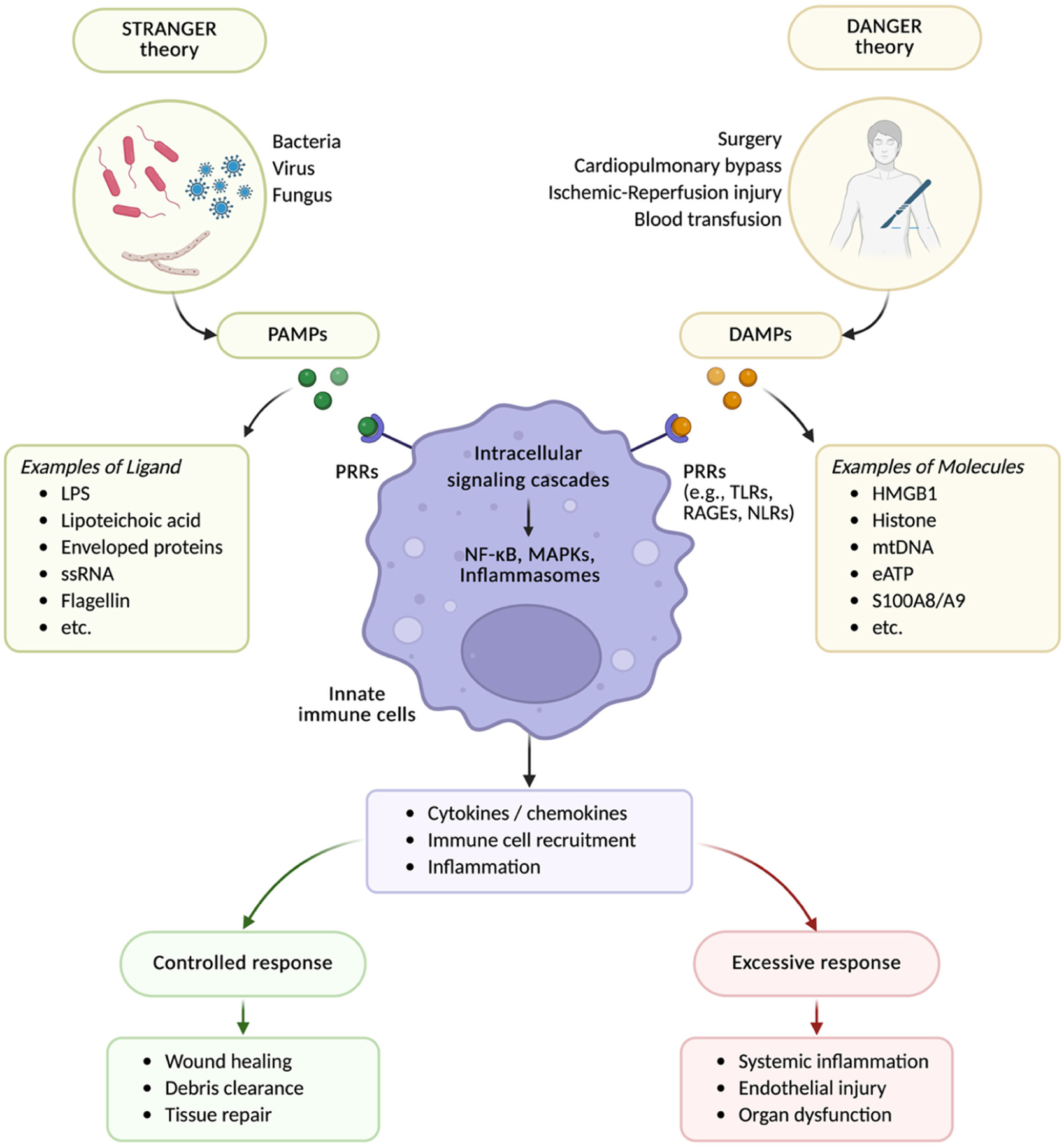
Recognition of pathogen-associated molecular patterns (PAMPs) and damage-associated molecular patterns (DAMPs) by the innate immune system. The innate immune system integrates signals from both exogenous (PAMPs) and endogenous (DAMPs) ligands through PRRs. PAMPs, such as lipopolysaccharide (LPS), lipoteichoic acid, flagellin, and viral RNA, arise from invading microorganisms (stranger model). DAMPs, including HMGB1, histones, mitochondrial DNA (mtDNA), extracellular ATP (eATP), and S100A8/A9, are released during sterile injury such as surgery, ischemia–reperfusion, cardiopulmonary bypass, and blood transfusion (danger signals). PRRs (e.g., Toll-like receptors, NOD-like receptors, RAGE) activate intracellular signaling cascades (e.g., MyD88, MAPK), leading to cytokine and chemokine release, immune cell recruitment, and amplification of inflammation. Controlled responses facilitate wound healing, debris clearance, and tissue repair, whereas excessive or dysregulated responses cause systemic inflammation, endothelial injury, and organ dysfunction. Explanation in the text. Created in BioRender. Maisat, W. (2025) https://BioRender.com/skorejb.

Recognition of DAMPs occurs through PRRs, a broad family of innate immune sensors. These receptors are expressed on immune cells as well as on endothelial and parenchymal cells, enabling both systemic and tissue-specific responses [24]. Their physiological role is to detect danger signals, whether derived from invading microbes or from injured host tissues. In the perioperative setting, the most relevant PRR families are Toll-like receptors (TLRs), the receptor for advanced glycation end products (RAGE), and NOD-like receptors (NLRs). Engagement of these receptors activates intracellular signaling cascades. For TLRs, this involves recruitment of the adaptor protein myeloid differentiation primary response 88 (MyD88), which activates downstream pathways including nuclear factor kappa B (NF-κB) and the mitogen-activated protein kinases (MAPKs). RAGE also activates MAPKs and NF-κB through distinct adaptors. RAGE also helps to mobilize some DAMPs to the intracellular space and present to TLRs. Once activated, NF-κB translocates to the nucleus, while MAPKs stimulate transcription factors such as activator protein-1 (AP-1). Together, these promote the transcription of pro-inflammatory cytokines, chemokines, and adhesion molecules that sustain inflammation (Figure 2). In addition, certain DAMPs contribute to inflammasome activation. For example, high mobility group box 1 (HMGB1) can enhance NOD-like receptor protein 3 (NLRP3) priming through NF-κB signaling, while extracellular adenosine triphosphate (ATP) engages P2X7 receptors and mitochondrial stress releases reactive oxygen species (ROS) and mitochondrial DNA (mtDNA), all of which promote inflammasome assembly. The resulting caspase-1 activation drives interleukin (IL)-1β and IL-18 maturation and pyroptotic cell death, further amplifying inflammation and DAMP release.

### 3.3. Dual Roles of DAMPs: Healing and Harm

DAMPs play a dual role in the host response to tissue injury. At physiological levels, they locally serve as an important alarm signal that helps initiate healing. By activating the innate immune system, DAMPs promote leukocyte recruitment, clearance of cellular debris, and tissue regeneration. These local responses are essential for recovery after surgery or trauma, and in this context, DAMPs contribute to tissue repair rather than harm the host (Figure 2).

However, when DAMP release is excessive, sustained, or dysregulated, the same signals that support healing can become pathogenic. Instead of resolving local injury, they may drive persistent systemic inflammation, damage the vascular endothelium, disrupt coagulation, and impair organ function. These systemic effects can occur even when the initial insult is localized. For example, sterile tissue injury from surgical trauma or limb ischemia–reperfusion can trigger distant organ dysfunction through DAMP-mediated pathways.

## 4. Cell Death as a Source of DAMPs

**Figure 3.**
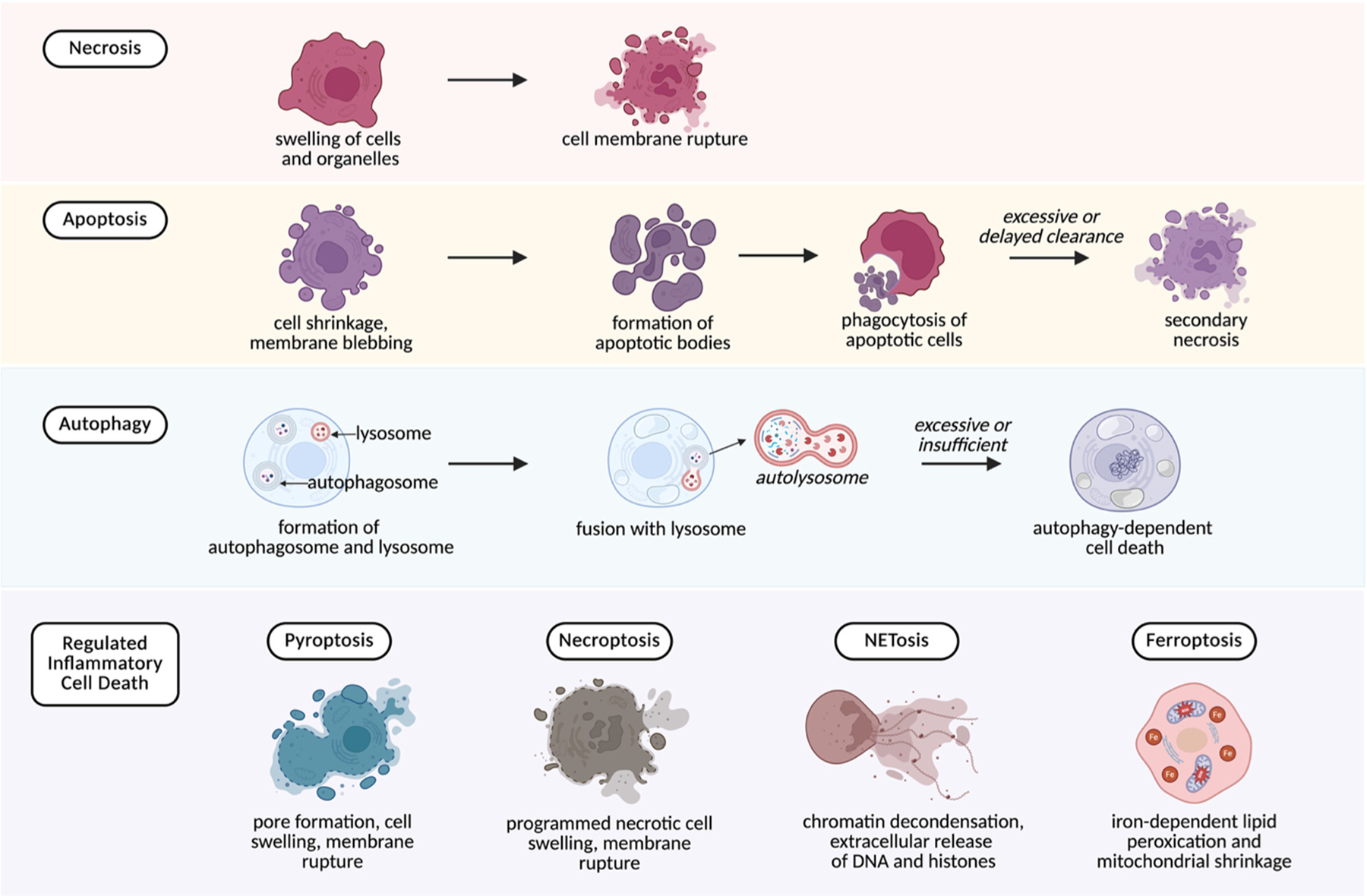
Overview of major cell death pathways and their characteristic morphological features. Necrosis involves early swelling of cells and organelles followed by rupture of the plasma membrane. Apoptosis progresses through cell shrinkage and membrane blebbing, formation of apoptotic bodies, and phagocytosis by neighboring cells. When clearance is delayed or insufficient, apoptotic cells may undergo secondary necrosis. Autophagy is characterized by formation of an autophagosome that fuses with a lysosome to generate an autolysosome. Excessive or inadequate autophagy may lead to autophagy-dependent cell death. The lower panel illustrates forms of regulated inflammatory cell death. Pyroptosis features pore formation, cell swelling, and membrane rupture. Necroptosis is marked by programmed necrotic swelling and membrane rupture. NETosis involves chromatin decondensation and extracellular release of DNA and histones. Ferroptosis is defined by iron-dependent lipid peroxidation and mitochondrial shrinkage. These regulated pathways differ in triggers and mechanisms but share the potential to generate DAMPs that propagate inflammatory responses. Explanation in the text. Created in BioRender. Maisat, W. (2025) https://BioRender.com/sij8drd.

The type of cell death that occurs during surgical injury strongly influences the nature and quantity of DAMPs released. Some modes of death are relatively silent, while others provoke robust inflammatory responses that can contribute to perioperative complications. Figure 3 illustrates types of cell death and their pathological feature.

- Necrosis
  Necrosis is an uncontrolled form of cell death that results from direct trauma, ischemia, or toxic injury. It is characterized by cellular swelling and membrane rupture, which cause the release of nuclear, mitochondrial, and cytosolic components into the extracellular space [25]. Necrosis is therefore a major contributor to DAMP release during surgical dissection and manipulation, liberating molecules such as HMGB1, ATP, histones, heat shock proteins (HSPs), mtDNA, and cell-free DNA (cfDNA) [26].
- Apoptosis
  Apoptosis is a tightly regulated process that normally avoids inflammation. Cellular content are packaged into apoptotic bodies that are cleared by phagocytes without DAMP release [27]. However, if clearance is delayed or overwhelmed, apoptotic cells progress to secondary necrosis, resulting in the release of DAMPs, particularly, HMGB1 [28]. Clinically, it has been shown that the rate of apoptosis is significantly increased in patients undergoing surgical stress [29].
- Autophagy-related death
  Autophagy is usually a protective mechanism that helps cells survive stress by removing damaged cytoplasmic components and preserving cellular homeostasis. In the perioperative setting, this function can reduce the release of DAMPs during tissue stress. However, when stress is excessive or prolonged, autophagy may fail and progress to cell death, allowing intracellular material to escape and contribute to extracellular pool of DAMPs [30].
- Regulated inflammatory cell death
  This group of programmed cell death pathways differs from apoptosis in that it provokes inflammation rather than suppressing it. Several regulated inflammatory cell death mechanisms are increasingly recognized as important in perioperative injury:
  - *Pyroptosis*—a pro-inflammatory form of cell death driven by NLRP3 inflammasome activation. A common trigger is extracellular ATP acting on P2X7 receptors. The process results in membrane rupture and the release of cytokines (IL-1β, IL-18) together with DAMPs, amplifying inflammation [31].
  - *Necroptosis*—a regulated form of cell death that resembles necrosis in its outcome but proceeds though a regulated signaling cascade. It culminates in membrane disruption and extracellular release of HMGB1, ATP, and S100 proteins [32].
  - *Ferroptosis*—associated with iron overload and lipid peroxidation, this process releases DAMPs such as HMGB1 and has been implicated in ischemia–reperfusion injury [33].
  - *NETosis*—a neutrophil-specific pathway in which neutrophils release neutrophil extracellular traps (NETs) composed of DNA, histones, and granule proteins. Although NETs immobilize pathogens, they are also rich in DAMPs that promote endothelial injury, thrombosis, and organ dysfunction [34]. NETosis has also been implicated in complications such as transfusion-related lung injury and thromboinflammation [35,36].

## 5 Perioperative Sources of DAMPs

Cell death processes become particularly relevant in the perioperative setting, where surgical and anesthetic interventions often create conditions that favor their activation. Necrosis, apoptosis, necroptosis, and other inflammatory cell death can be triggered by direct tissue injury, ischemia–reperfusion (I/R) injury, extracorporeal circulation, transfusion, and mechanical stress. Each of these perioperative events promotes the release of distinct DAMPs, which in turn contribute to systemic inflammation and postoperative complications.

### 5.1. Surgical Trauma

Surgical incision, dissection, and cauterization inevitably disrupt cells and extracellular structures. This causes the release of nuclear protein, cytosolic enzymes, mitochondrial component, and extracellular matrix fragments. While a controlled inflammatory response is necessary for wound healing, extensive surgical trauma can generate excessive release and propagate systemic inflammation. This mechanistic link provides a biological explanation for the higher inflammatory burden and complication rates seen with longer or more invasive procedures.

### 5.2. Ischemia–Reperfusion Injury

Temporary interruption of blood flow is common in vascular, transplant, and cardiac surgery, as well as during the use of tourniquets. In the ischemic phase, oxygen and nutrient deprivation deplete ATP stores, impair ion gradients, and promote mitochondrial dysfunction. These changes initiate necrosis and apoptosis in some cells, while others undergo necroptosis if the insult is severe or prolonged [37]. Reperfusion, although restoring circulation, paradoxically worsens injury by generating ROS, which destabilize cell membranes and activate regulated inflammatory cell death pathways such as pyroptosis and ferroptosis [38]. These processes release DAMPs including HMGB1, mtDNA, ATP, and HSPs [37,39], which amplify sterile inflammation beyond the ischemic tissue and potentially contribute to perioperative complications such as ALI, myocardial dysfunction, and AKI, even after otherwise successful operations.

### 5.3. Cardiopulmonary Bypass and Extracorporeal Circulation

CPB is among the most intense stimuli for perioperative DAMP release because it exposes blood to artificial surfaces and non-physiological flow conditions. Blood contact with artificial surfaces and non-physiological flow conditions activates leukocytes, platelets, and complement, while ischemia–reperfusion of the myocardium and other organs adds further injury [40]. High circulating concentrations of HMGB1, histones, S100A8/A9, and HSPs have been consistently documented during and after CPB [34,41]. The magnitude of this response correlates with bypass duration and ischemic severity, providing a mechanistic rationale for the poorer outcomes seen after prolonged CPB and the relative advantages reported with off-pump coronary artery bypass (OPCAB) surgery. The frequent need for perioperative transfusion further compounds this DAMP burden.

### 5.4. Blood Product Transfusion

Blood products accumulate structural and biochemical changes during storage, known as “storage lesions” [42], which generate bioactive molecules such as heme, cfDNA, extracellular ATP, HMGB1, and microparticles [11,43,44]. These changes occur not only in red cells but also in plasma-rich components such as platelets and fresh frozen plasma [11]. When transfused, these molecules enter the circulation and activate PRRs on immune and endothelial cells. In the pulmonary vasculature, this process primes neutrophils, leading to adhesion, activation, and the release of ROS and proteolytic enzymes. The resulting endothelial injury and increased vascular permeability form the basis of transfusion-related acute lung injury (TRALI) [11]. Transfusion may also induce broader immune dysregulation, referred to as transfusion-related immunomodulation (TRIM) [45]. These insights provide a mechanistic explanation for why restrictive transfusion thresholds and patient blood management (PBM) strategies improve outcomes.

### 5.5. Mechanical Ventilation

Mechanical stress is another iatrogenic source of DAMPs. High tidal volumes and excessive airway pressures stretch alveoli, disrupting epithelial and endothelial barriers, and release of extracellular ATP and HSP70 [46,47]. These DAMPs activate immune pathways that promote cytokine production, leukocyte recruitment, and pulmonary inflammation, potentially contributing to ventilator-induced lung injury (VILI) [48]. Importantly, this inflammatory reaction is not confined to the lungs. Systemic spillover of inflammatory mediators may initiate and propagate systemic inflammation and multi-organ dysfunction [47]. Protective ventilation strategies mitigate these effects and have become standard practice in perioperative and critical care.

## 6. Pathophysiological Consequences of DAMP Release

The perioperative environment provides numerous triggers for DAMP release. These molecules do not remain confined to the site of injury but spill into the circulation, spread systemically, and interact with immune systems in ways that amplify inflammation and disrupt homeostasis. The downstream consequences include endothelial injury, coagulation disturbances, propagation of systemic inflammation, and ultimately organ dysfunction. Taken together, these processes form the pathophysiological bridge between intraoperative events and postoperative complications.

### 6.1. Endothelial Activation and Microvascular Dysfunction

The vascular endothelium is one of the earliest and most vulnerable targets of circulating DAMPs. Through PRR-mediated signaling [49], DAMPs disrupt endothelial barrier integrity, increase permeability, and induce the expression of adhesion molecules such as intercellular adhesion molecule-1 (ICAM-1) and vascular cell adhesion molecule-1 (VCAM-1) [50]. These changes promote leukocyte adhesion and transmigration into the site of injury [51].

Loss of barrier function results in capillary leak and tissue edema, while reduced nitric oxide (NO) bioavailability compromises vasodilation and microvascular perfusion [52]. Endothelial activation also promotes a shift toward a procoagulant phenotype, with increased tissue factor (TF) exposure and von Willebrand factor (vWF) release [53]. Collectively, these responses impair vascular homeostasis, reduce oxygen delivery, and predispose to postoperative inflammatory complications.

### 6.2. Thrombosis and Coagulopathy

Perioperative release of DAMPs exerts complex effects on hemostasis, with most molecules driving immunothrombosis while others exert anticoagulant influences.

On the prothrombotic side, DAMPs may act at multiple stages of coagulation. HMGB1 initiates clotting by upregulating TF expression on endothelial cells and inhibiting the protein C pathway [54]. mtDNA propagates the cascade through activation of the intrinsic pathway [55], while S100A9 enhance thrombus formation [56]. Clot stability is reinforced by histone-induced secretion of plasminogen activator inhibitor-1 (PAI-1) [57], which suppresses fibrinolysis, and by cfDNA, which impairs plasmin-mediated fibrin degradation [58]. In addition, DAMPs promote platelet activation and drive neutrophils to form extracellular traps (NETs), providing a structural scaffold for thrombin generation, fibrin deposition, and further platelet aggregation [36]. Together, these processes create a potent prothrombotic environment that can escalate into widespread microvascular thrombosis.

Excessive activation, however, may culminate in consumptive coagulopathy, where sustained thrombin generation and fibrin deposition deplete platelets and coagulation factors. Clinically, this manifests as disseminated intravascular coagulation (DIC), characterized by the paradoxical coexistence of microvascular thrombosis and bleeding.

In contrast, certain DAMPs can exert anticoagulant effects. Heparan sulfate has been associated with impaired thrombin generation in severely injured trauma patients [59], while elevated serum histone H3 levels were observed in septic patients with coagulopathy and predictive of their higher mortality risk [60]. Although these findings may reflect protective or compensatory mechanisms in isolation, their coexistence with strong prothrombotic signals contributes to the unstable hemostatic balance that characterizes perioperative critical illness.

### 6.3. Propagation of Systemic Inflammation

DAMP release initiates and sustains systemic inflammation through complex signaling pathways and cellular interactions. Activation of PRRs leads to production of pro-inflammatory cytokines and chemokines, such as tumor necrosis factor-α (TNF-α), interleukin (IL)-1β, IL-6, IL-8, and interferons. These mediators recruit and activate leukocytes and establish a positive feedback loop that promotes further DAMP release.

Certain DAMPs also stimulate neutrophils to undergo NETosis, generating extracellular traps rich in DNA, histones, and HMGB1 that act as additional DAMPs and sustain inflammation. Activation of the NLRP3 inflammasome further enhances secretion of IL-1β and IL-18 through pyroptosis, intensifying the systemic response. Collectively, these processes perpetuate sterile inflammation that can progress to SIRS and multi-organ dysfunction in susceptible patients.

### 6.4. Organ Dysfunction

The convergence of endothelial injury, coagulation disturbances, and sustained inflammation drives organ dysfunction. Capillary leak and microvascular thrombosis reduce oxygen and nutrient delivery, while mitochondrial dysfunction and oxidative stress impair cellular energy metabolism. NETs exacerbate this by obstructing capillary flow and propagating inflammation.

These mechanisms manifest clinically as ALI, acute respiratory distress syndrome (ARDS), AKI, myocardial dysfunction, DIC, or perioperative neurocognitive disorder (PND). Although the affected organ may differ, the underlying mechanism is unified, driven by sterile inflammation initiated by DAMP release. This model provides a coherent explanation for how apparently routine intraoperative events can progress to systemic complications and highlights the potential role of DAMPs in perioperative outcomes. Table 2 summarizes the major DAMPs implicated in perioperative organ dysfunction, their pathways, and associated clinical consequences.

## 7. Anesthetic Implications

Many perioperative events act as potent triggers for DAMP release, and anesthesia is closely intertwined with these processes. This positions anesthesiologists to influence not only immediate physiological stability but also the molecular signals that shape postoperative recovery. Unlike cytokines, which represent downstream mediators common to many inflammatory cascades, DAMPs function as upstream initiators of sterile inflammation. Strategies that reduce their release or limit their recognition therefore represent opportunities to interrupt the inflammatory cascade at an earlier and potentially more effective stage.

### 7.1. Anesthetic Agents

Experimental and early clinical evidence suggests that anesthetic agents may modulate DAMP release and signaling These effects are not yet strong enough to dictate practice guideline, but they may provide biological rationale for tailoring drug selection in high-risk settings. Table 3 provides an overview of current evidence on anesthetic agents and their potential interactions with DAMP biology.

Volatile anesthetics such as sevoflurane and isoflurane have shown mixed results. In experimental models, they can reduce HMGB1 release and attenuate I/R injury [98], but prolonged exposure has also been associated with impaired cognitive outcomes [125], particularly in vulnerable brains [97]. These discrepancies may be due to differences in study conditions, including the duration and depth of anesthesia, timing of biomarker collection, baseline inflammatory status, or tissue-specific susceptibility.

In contrast, propofol has demonstrated more consistent protective effects in preclinical studies, including reduced mtDNA and HMGB1 release, suppression of HMGB1-driven pathways, and reduction in oxidative stress. Clinical evidence is less consistent. For example, in posterior spinal fusion surgery, isoflurane anesthesia was associated with higher perioperative mtDNA levels compared with propofol, suggesting greater DAMP release [126]. In contrast, in a pediatric liver transplantation study reported similar rises in IL-6, TNF-α, and HMGB1 with both propofol and sevoflurane, with no clear difference between groups [127].

Among available agents, dexmedetomidine (DEX) has been studied most extensively for its anti-inflammatory potential. Experimental data show that DEX can suppress HMGB1 release, inhibit TLR4 activation, and reduce pyroptosis, necroptosis, and I/R injury. Clinical studies suggest that DEX reduces the incidence of PND, although this benefit may involve multiple pathways beyond HMGB1 alone. It is worth noting that some anesthetics do not directly alter DAMP molecules themselves but instead act by inhibiting TLR signaling, thereby limiting the immune system’s ability to amplify DAMP-driven inflammation.

### 7.2. Transfusion Practice

Blood products represent an important iatrogenic source of DAMPs. Recognition that stored blood products contain DAMPs reinforces the importance of PBM principles in anesthetic practice. Restrictive transfusion thresholds should be employed whenever possible, and unnecessary transfusions should be avoided. When transfusion is unavoidable, strategies such as using fresher units, preferring leuko-reduced or washed products, and minimizing exposure to plasma-rich components can reduce the DAMP burden. Careful intraoperative hemostasis, antifibrinolytic therapy, and blood conservation techniques further decrease reliance on transfusion. These measures, already advocated by PBM guidelines, gain additional mechanistic support from DAMP biology and highlight the anesthesiologist’s role in actively mitigating transfusion-related complications.

### 7.3. Cardiopulmonary Bypass and Extracorporeal Circuits

In the setting of CPB, strategies that minimize the inflammatory burden should be prioritized. Several strategies may attenuate this response. Minimizing bypass duration and using shorter or simplified circuits reduces the blood–surface interface and the extent of hemolysis. Optimizing perfusion parameters and oxygen delivery helps limit I/R injury, while biocompatible circuit coatings reduce leukocyte and platelet activation. Ultrafiltration during CPB can remove circulating inflammatory mediators, and newer approaches such as cytokine adsorption devices have shown promise in selectively reducing both cytokines and DAMP-related mediators from the circulation [128].

### 7.4. Ischemia–Reperfusion Injury

I/R injury is encountered not only in cardiac and vascular surgery but also in transplantation, abdominal procedures, and operations using tourniquets. The magnitude of injury can be influenced by perioperative management. Key strategies include minimizing the duration of ischemia, maintaining adequate perfusion and oxygen delivery, and avoiding sustained hypotension at critical periods. Experimental approaches such as ischemic preconditioning, postconditioning, and volatile anesthetic preconditioning have shown potential but remain controversial, with inconsistent results in clinical trials. At present, the most reliable preventive measures remain careful intraoperative monitoring, timely restoration of blood flow, and optimization of hemodynamics to reduce the burden of reperfusion-related inflammation.

### 7.5. Monitoring and Risk Stratification

Current perioperative risk assessment relies primarily on clinical evaluation and physiologic monitoring. However, DAMPs are increasingly recognized as potential biomarkers of perioperative risk. Elevated levels of HMGB1, S100 proteins, mtDNA, and histones have been associated with postoperative organ dysfunction and often precede clinical deterioration. Although measurement of these biomarkers is not yet part of routine practice, ongoing research suggests that they could contribute to earlier detection of complications and more refined risk stratification [34].

For example, in patients undergoing surgical aortic valve replacement with cardiopulmonary bypass, plasma mtDNA levels increased more than tenfold and were associated with postoperative bleeding, hepatic dysfunction, and prolonged hospitalization [129]. Similarly, postoperative increases in circulating mtDNA were correlated with the development of systemic inflammatory response syndrome and multiple organ dysfunction following pancreaticoduodenectomy [130]. Even though specific cutoffs remain undefined for clinical application, these studies demonstrate the potential of perioperative DAMP levels as biomarkers for monitoring inflammatory stress and improving risk stratification.

## 8. Future Directions

Much of the current knowledge on DAMPs comes from in vitro experiments and preclinical animal models. These studies have provided valuable mechanistic insights, but their direct translation to the clinical perioperative setting remains challenging. The complexity of surgical care, patient heterogeneity, and the interplay of multiple overlapping inflammatory pathways limit the extrapolation of experimental findings. Moreover, clinical studies measuring perioperative DAMPs are often small, methodologically heterogeneous, and rely on non-standardized assays, making cross-study comparisons difficult.

Future research should aim to bridge these gaps by clarifying how DAMP biology can move from mechanistic insight to clinical application. Large, well-designed studies are needed to validate perioperative DAMPs as reliable biomarkers for risk stratification and early detection of complications. Interventions that target DAMP release, signaling, or clearance, such as pharmacologic inhibitors, extracorporeal adsorption techniques, or tailored anesthetic regimens, hold promise but require rigorous clinical evaluation. Ultimately, integrating DAMP biology into perioperative care may enable more personalized anesthetic strategies, reduce postoperative morbidity, and strengthen the role of anesthesiologists in advancing perioperative inflammation and recovery science.

## 9. Conclusions

DAMPs represent upstream mediators that bridge perioperative cellular injury to systemic inflammation and organ dysfunction. Recognizing their role reframes postoperative complications not only as sequelae of hemodynamic or infectious events but also as consequences of sterile inflammation triggered by surgical and anesthetic stress.

For anesthesiologists, this perspective provides a mechanistic rationale for existing practices such as protective ventilation, restrictive transfusion strategies, and careful ischemia–reperfusion management, while also highlighting emerging opportunities for innovation in monitoring and intervention. Translating DAMP biology into perioperative care requires rigorous clinical validation, standardized measurement approaches, and integration with established risk stratification tools. Ultimately, embedding DAMPs into perioperative practice may facilitate more personalized anesthetic management and improved postoperative outcomes.

## Figures and Tables

**Table 1.** Major perioperative damage-associated molecular patterns (DAMPs).

Location/Source	Acronym	Full Name	Function (Normal)	PRRs/Recognition Pathways	References
**Nuclear**	HMGB1	High-Mobility Group Box 1	Chromatin binding; transcriptional regulation	TLR2, TLR4, RAGE	[12,13]
	Histones	Histone proteins	Structural organization and stability of chromatin	TLR2, TLR4, TLR9; direct endothelial binding	[14]
	Extracellular DNA	Nuclear DNA (cfDNA)	Genetic material	TLR9, cGAS–STING	[15,16]
**Mitochondrial**	mtDNA	Mitochondrial DNA	Encodes mitochondrial proteins	TLR9, cGAS–STING, NLRP3	[17]
	ATP	Adenosine triphosphate	Energy currency of the cell	P2X7; NLRP3 inflammasome	[18]
	N-formyl peptides	Mitochondrial peptides	Chemotactic signals	FPR-1	[19]
**Cytosolic**	S100A8/A9	S100 calcium-binding proteins A8/A9	Stress response; intracellular signaling	RAGE, TLR4	[20]
	S100A12	S100 calcium-binding protein A12	Proinflammatory mediator	RAGE, TLR4	[10]
	HSPs	Heat-shock proteins	Molecular chaperones; protein folding	TLR2, TLR4	[10]
	UA	Uric acid	End product of purine metabolism	NLRP3 inflammasome	[21]
**Extracellular matrix (ECM)**	Biglycan	Small leucine-rich proteoglycan	Structural support; released upon ECM damage	TLR2, TLR4	[22]
	LMW-HA	Low molecular weight hyaluronan	ECM degradation fragment	TLR2, TLR4	[22]
	Heparan sulfate	Glycosaminoglycan fragments	ECM component; endothelial injury signal	TLR4	[23]
**Blood/Transfusion-related**	Free heme	Cell-free heme	Oxygen transport; pro-oxidant when released	TLR4	[11]
	Lipid peroxidation products	n/a	n/a	TLR4	[11]
	Microparticles	Platelet- or FFP- or RBC-derived vesicles	n/a	NLRP3	[11]

**Abbreviations:** cfDNA, cell-free DNA; ECM, extracellular matrix; FPR-1, formyl peptide receptor-1; LMW-HA, low molecular weight-hyaluronan; NLRP3, NOD-like receptor family pyrin domain-containing 3; PRR, pattern recognition receptor; RAGE, receptor for advanced glycation end products; TLR, Toll-like receptor.

**Table 2.** DAMPs implicated in perioperative organ dysfunction, their triggers, signaling pathways, and supporting evidence from clinical and preclinical studies.

Clinical Manifestation	DAMPs Involved	Triggers	Mechanisms/Pathways	Clinical/Experimental Relevance	Evidence Type
**ALI/ARDS**	**mtDNA**	Sepsis	n/a	Elevated plasma mtDNA in septic patients with ALI was associated with higher mortality.	Clinical observation [61]
		Lung I/R injury	GSDMD-mediated pores promote mtDNA leakage into neutrophils, activating cGAS-STING and inducing NET formation	NET release driven by mtDNA contributed to lung injury during I/R.	Preclinical (mice) [62]
	**MTD**	MTD injection	Activates NLRP3, FPR-1, and TLR9 Increases Ca^2^+ flux and MAPK activation in neutrophils	MTD promoted alveolar macrophage activation and acute lung injury.	Preclinical (mice) [63,64]
	**HGMB1**	Postoperative mechanical ventilation	n/a	Elevated HMGB1 detected in BALF of ventilated patients without preexisting lung injury.	Clinical RCT [65]
		CPB	NLRP3/ASC-mediated alveolar macrophage pyroptosis TLR4 activation	Elevated HGMB1 secretion in serum and BALF Anti-HMGB1 treatment reduced ALI after CPB. and lowered IL-1β, TNF-α, HGMB1, and TLR4 expression in lung tissue.	Preclinical (rat) [66,67]
		Intestinal I/R	MyD88-mediated NET formation	HMGB1-induced NETosis exacerbated intestinal I/R-associated ALI. NET degradation and inhibition mitigated injury.	Preclinical (mice) [68]
	**Histones**	LPS-induced SIRS/ARDS	Induce pulmonary endothelial activation via TLRs, promoting neutrophil adhesion and activation	Histones act as potent proinflammatory mediators in ARDS.	Preclinical (mice) [69]
		Lung I/R (lung transplantation)	Stimulate CTSC secretion from alveolar macrophages, triggering NETosis via NADPH oxidase-derived ROS	Increased NETosis observed in lung transplant patients with primary graft dysfunction. CTSC-inhibition attenuated NETosis and I/R injury in mice.	Clinical case–control; preclinical (mice) [70]
	**S100A12**	Pneumonia with ARDS	RAGE activation	S100A12 and RAGE are highly expressed in lung tissue during ALI. BALF levels of S100A12 and sRAGE are elevated in ARDS patients. S100A12 induces a proinflammatory response in lung endothelial cells in vitro.	Clinical case–control; experimental [71]
	**S100A9**	LPS-induced SIRS/ALI	NLRP3 activation	S100A9 is upregulated in lung tissue; blockade attenuated inflammation and apoptosis.	Preclinical (mice) [72]
	**Extracellular ATP**	Mechanical ventilation	P2Y1 receptor activation	Extracellular ATP in BALF and P2Y1 receptor expression in lung tissues are increased after mechanical ventilation (high tidal volume > low tidal volume > sham ventilation).	Preclinical (mice) [46]
**Myocardial dysfunction**	**Histones**	Sepsis	n/a	Circulating histone levels correlated with cTn levels in septic patients and mediate cardiomyocyte injury ex vivo. High histone levels were associated with new-onset LV dysfunction and arrhythmias. Anti-histone intervention attenuated cardiac injury and dysfunction in septic models.	Clinical case–control; preclinical (mice) [73]
		Global I/R injury	Direct cytotoxicity to cardiomyocytes through a mechanism independent of TLR4 and NF-kB signaling	Circulating histones increased following I/R injury; histone H4 levels positively correlated with infarct size.	Preclinical (rat) [74]
	**HMGB1**	Myocardial I/R	Endothelial HMGB1-AIM2 axis regulating endothelial pyroptosis	Elevated HMGB1 detected in cardiac tissue and circulation after I/R. Endothelial HMGB1 knockout preserved function by reducing infarct size, maintaining barrier integrity, and attenuating inflammation and oxidative stress.	Preclinical (murine) [75]
		Myocardial I/R	JNK/ERK/NF-κB and RAGE signaling activation	HMGB1 levels increased within 30 min after hypoxia in vitro, and during ischemic myocardial injury in vivo. Inhibition of HMGB1 reduced infarct size and tissue damage.	Preclinical (mice) [76]
		Myocardial I/R	HMGB1/TLR4 signaling	TLR4 mutation reduced cardiomyocyte apoptosis and infarct size after myocardial I/R.	Preclinical (mice) [77]
	**S100A8/A9**	Myocardial I/R, PCI	Suppresses mitochondrial energy production by inhibiting complex I via TLR4/ERK signaling, leading to downregulation of PGC-1α/NRF1 pathways	Serum S100A8/A9 levels were significantly increased one day after PCI. Higher levels were associated with the incidence of MACEs.	Clinical observation; preclinical (mice) [78]
**POAF**	**mtDNA**	Cardiac surgery with CPB	n/a	Patients with POAF had higher pericardial mtDNA levels compared with those without.	Clinical observation [17,79]
	**HMGB1**	Cardiac surgery with CPB	n/a	Elevated HMGB1 was associated with increased susceptibility to POAF after CABG with CPB.	Clinical observation [80]
**AKI**	**HGMB1**	Renal I/R	HMGB1/TLR4 signaling	HMGB1 promoted renal damage after I/R injury. Administration of neutralizing Ab to HMGB1 before or soon after I/R protected against AKI	Preclinical (mice) [81]
		Renal I/R	n/a	HMGB1 expression progressively increased in ischemic kidneys. HMGB1 neutralization preserved renal function and reduced tubular necrosis	Experimental; preclinical (mice) [82]
	**mtDNA**	Surgical critical illness	n/a	Urinary mtDNA correlated with markers of renal injury/dysfunction. Elevated levels identified new-onset AKI and predicted RRT need and hospital mortality.	Clinical observation [83]
		Cardiac surgery with CPB	n/a	Urinary mtDNA was elevated in progressive AKI and was associated with worsening severity	Clinical observation [84]
	**Histone**	Renal I/R	NET formation	Histone secreted from ischemic tubular cells primed neutrophils to form NETs in vitro. NETs were observed in kidney biopsies from patients with post-transplant ATN after prolonged cold ischemic time. Circulating histones promoted lung injury and remote organ dysfunction after renal I/R.	Clinical observation; preclinical (mice); experimental [85]
**Liver**	**HMGB1**	Hepatic I/R	HMGB1/TRL4 signaling	HMGB1 expression was upregulated in hepatocytes after hypoxia in vitro and in the liver after I/R in vivo. Neutralizing HMGB1 reduced inflammatory mediators and protected against hepatic I/R injury.	Experimental; preclinical [86]
**Coagulopathy**	**Histone**	Sepsis	n/a	Serum histone H3 levels were elevated in septic patients with coagulopathy and predicted a higher risk of mortality.	Clinical observation [60]
	**Heparan sulfate**	Trauma	n/a	Heparan sulfate was associated with impaired thrombin generation.	Clinical observation [59]
**DIC**	**Histone**	Histone injection	Histones bind platelets, induce calcium influx, and recruit adhesion molecules to promote platelet aggregation.	Histone–platelet interaction contributed to thrombosis in preclinical models.	Preclinical (mice) [87]
	**Histone**	Sepsis Histone injection	Extracellular histones trigger platelet aggregation and microvascular occlusion.	Histone H3 was markedly elevated in septic patients with DIC but absent in healthy controls. Extracellular histones caused massive thromboembolism consumptive coagulopathy in mice.	Clinical case–control; preclinical [34,88]
	**HMBG1**	Sepsis	n/a	Patients with DIC had significantly elevated plasma HMGB1. HMGB1 levels correlated with DIC score and SOFA score.	Clinical observation [89]
**PND (POD/POCD)**	**HMGB1**	Major GI surgery	n/a	Serum HMGB1 levels were elevated in elderly patients and associated with postoperative cognitive decline	Clinical [90]
		Splenectomy	HMGB1/RAGE signaling disrupts the blood–brain barrier and promotes neuroinflammation.	Elevated HMGB1 and RAGE expression were observed with evidence of blood–brain barrier disruption and cognitive impairment	Preclinical (rat, mice) [91,92]
	**mtDNA**	Prolonged anesthesia with sevoflurane	Microglial NLRP3 inflammasome activation cGAS-STING signaling	Prolonged sevoflurane anesthesia triggered cognitive dysfunction and neurological impairment in mice	Preclinical (mice) [93]
	**S100A8/A9**	Tibial fracture surgery	TLR4/MyD88 signaling promotes neuroinflammation and microgliosis	S100A8/A9 expression was elevated in PBMCs and hippocampus after surgery. Antibody blockade reduced microgliosis and improved cognitive function 24 h after surgery.	Preclinical (mice) [94]

**Abbreviations:** ALI, acute lung injury; ARDS, acute respiratory distress syndrome; AIM2, absent in melanoma 2; AKI, acute kidney injury; ATN, acute tubular necrosis; BALF, bronchoalveolar lavage fluid; CABG, coronary artery bypass grafting; cGAS, cyclic GMP–AMP synthase; CPB, cardiopulmonary bypass; CTSC, cathepsin C; DAMP, damage-associated molecular pattern; DIC, disseminated intravascular coagulation; ERK, extracellular signal-regulated kinase; GSDMD, gasdermin D; HMGB1, high-mobility group box 1; IL, interleukin; I/R, ischemia–reperfusion; JNK, c-Jun N-terminal kinase; LV, left ventricle; MACE, major adverse cardiac event; MAPK, mitogen-activated protein kinase; mtDNA, mitochondrial DNA; NET, neutrophil extracellular trap; NF-κB, nuclear factor kappa B; NLRP3, NOD-like receptor family pyrin domain-containing 3; PBMC, peripheral blood mononuclear cell; PCI, percutaneous coronary intervention; PND, perioperative neurocognitive disorder; POD, postoperative delirium; POCD, postoperative cognitive dysfunction; POAF, postoperative atrial fibrillation; RAGE, receptor for advanced glycation end products; RRT, renal replacement therapy; ROS, reactive oxygen species; SOFA, sequential organ failure assessment; TLR, Toll-like receptor.

**Table 3.** Preclinical evidence for anesthetic and adjunctive modulation of DAMP pathways.

Agent		DAMPs Affected		Pathways/Mechanisms		Reported Effects (Preclinical Study)
**Volatile agents**						
Sevoflurane		↑ mtDNA [93,95] ↑ HMGB1 [96,97] ↓ HMGB1 [98,99] ↑ HSP70 [100]		cGAS-STING-NLRP3 inflammasome activation [93,95] HMGB1-induced NLRP3/ASC activation [96,97] TLR4/MyD88/NF-κB and TRAF signaling [97–99]		**Protective effects:** Postcondition reduced hepatic, cerebral, and myocardial I/R injury, suppressed neuroinflammation [98–100]. **Adverse effects:** Prolonged or prenatal exposure associated with neuroinflammation, impaired cognition, and memory deficits [93,95–97].
Isoflurane		↑ HMGB1 [101] ↓ HMGB1 [102] ↑ HSP60 [103]		RAGE signaling [101] TLR2 and TLR4 signaling [102,103]		**Protective effects:** Reduced microglial activation and neuroinflammation [102,103] **Adverse effects:** May induce pyroptosis in lung cancer model [101].
**Intravenous agents**						
Propofol		↓ HMGB1 [104–107]		Inhibits TLR4/PKR [104] and HMGB1-NLRP3 inflammasome signaling [106] Reduces mitochondrial oxidative stress [105] Modulates miRNA-451/HMGB1 signaling [107]		**Protective effects:** Reduced pulmonary and myocardial injury in I/R models [104,106,107]; attenuated VILI [105].
Ketamine	↓ HMGB1 [108–112] ↑ mtDNA [113]		HMGB1-RAGE signaling, with effects on autophagy and microglial polarization [108] TLR4/MyD88/TRAF and NF-κB inhibition [109–112] cGAS-STING pathway [113]		**Protective effects:** Reduced LPS- and sepsis-induced lung injury [109–111]; inhibited HMGB1-induced endothelial inflammation [112]; exerted antidepressant effect [108]. **Adverse effects:** In specific setting (e.g., ketamine-induced cystitis in rodents), mitochondrial damage and mtDNA release may worsen inflammation [113].	
**Adjuncts**						
Dexmedetomidine (DEX)	↓ HMGB1 [114–120] ↑ HSPA12B [121]		Inhibits TLR4/MyD88/NF-κB and RAGE signaling [114–118] Suppresses NLRP3 inflammasome activation [119] Inhibit pyroptosis and necroptosis [120] Enhances protective HSPA12B expression through TLR4/NF-κB signaling [121]		**Protective effects:** Attenuated hippocampal neuroinflammation and PNDs [114]; reduced myocardial and renal injury from ischemia–reperfusion [115,116,119,120]; mitigated renal I/R injury and LPS-induced ALI [118,121]; alleviated neuropathic pain and inflammatory response induced by a chronic constriction injury [117]. **Clinical data:** DEX showed anti-inflammatory effects and reduced the incidence of PNDs, but HMGB1 levels were not significantly correlated with the incidence of PND in patients [122].	
Remimazolam	↓ HMGB1 [123]		Inhibits TLR4/NF-κB signaling [123]		**Protective effects:** Attenuated POCD after DHCA [123].	
**Opioids**						
Remifentanil	↓ HMGB1 [124]		Reduces NF-κB activation [124]		**Protective effects:** Anti-inflammatory effects in septic models [124].	

**Abbreviations:** ASC, apoptosis-associated speck-like protein containing a CARD; cGAS, cyclic GMP–AMP synthase; DHCA, deep hypothermic circulatory arrest; DEX, dexmedetomidine; HMGB1, high-mobility group box 1; HSPA12B, heat shock protein family A member 12B; I/R, ischemia–reperfusion; LPS, lipopolysaccharide; miRNA, microRNA; mtDNA, mitochondrial DNA; NF-κB, nuclear factor kappa B; NLRP3, NOD-like receptor family pyrin domain containing 3; PND, perioperative neurocognitive disorder; POCD, postoperative cognitive dysfunction; RAGE, receptor for advanced glycation end products; TLR, Toll-like receptor; TRAF, TNF receptor-associated factor; VILI, ventilator-induced lung injury. Upper arrow increase, lower arrow decreases.

## Data Availability

No new data were created or analyzed in this study. Data sharing is not applicable to this article.
